# Disrupting the Path from Depression to Loneliness: Multilevel Resilience among Older Sexual Minority Men with and without HIV

**DOI:** 10.1007/s10461-024-04416-w

**Published:** 2024-07-24

**Authors:** Maria De Jesus, Deanna Ware, Steven Meanley, Mark Brennan-Ing, Chukwuemeka N Okafor, Steve Shoptaw, Sabina Haberlen, Valentina Stosor, M. Reuel Friedman, Michael Plankey

**Affiliations:** 1 Department of Environment, Development and Health, School of International Service and Center on Health, Risk, and Society, American University, 4400 Massachusetts Avenue NW, Washington, DC 20016 USA; 2 Department of Medicine, Georgetown University Medical Center, Washington, DC USA; 3 Department of Family and Community Health, University of Pennsylvania School of Nursing, Philadelphia, USA; 4 Department of Geriatrics, Brookdale Center for Healthy Aging, City University of New York, New York City, NY USA; 5 Department of Public Health, Robbins College of Health and Human Sciences, Baylor University, Waco, USA; 6 Department of Family Medicine, University of California, Los Angeles, CA USA; 7 Department of Epidemiology, Bloomberg School of Public Health, Johns Hopkins, Baltimore, MD USA; 8 Department of Medicine, Feinberg School of Medicine, Northwestern University, Chicago, IL USA; 9 Department of Infectious Diseases and Microbiology, School of Public Health, University of Pittsburgh, Pittsburgh, PA USA; 10 Department of Medicine, Division of General Internal Medicine, Georgetown University, Washington, DC USA

**Keywords:** Depression, Loneliness, Resilience, Sexual minority men, Aging, HIV/AIDS, Depresión, Soledad, Resiliencia, Hombres de minorías sexuales, Envejecimiento, VIH/SIDA

## Abstract

Existing studies examining resilience among sexual minority men (SMM) have been limited by only analyzing 1 level of resilience. We therefore investigated the impact of multiple levels of resilience on the bidirectional relationship between loneliness and depression symptoms among older SMM. Loneliness, depression symptoms, and multilevel resilience scores were collected across 3 time points (October 2016 to March 2017 [T1]; October 2017 to March 2018 [T2]; and October 2018 to March 2019 [T3]) among 1,264 SMM aged 40 years and older living with and without HIV. Longitudinal mediation models were used to test the mediating effect of the multilevel resilience factors at T2 on the bidirectional relationship between loneliness and depression symptoms, adjusting for sociodemographic covariates. The multilevel resilience factors were negatively associated with loneliness and depression symptoms at T1. The individual-level global resilience factor was associated with decreased odds of depression symptoms (odds ratio [OR] = 0.50; 95% CI, 0.32–0.78), while the interpersonal-level relationship confidence (OR = 0.43; 95% CI, 0.24–0.77) and reliability (OR = 0.36; 95% CI, 0.15–0.84) factors were associated with decreased odds of loneliness at T3. The total effect of loneliness at T1 on depressive symptoms at T3 was β = 0.20 (95% CI, 0.11–0.28) and was reduced to β = 0.08 (95% CI, -0.04 to 0.20) after the inclusion of the multilevel resilience factors. The total effect of depressive symptoms at T1 on loneliness at T3 was similar (β = 0.21; 95% CI, 0.13–0.28) with the direct effect of β = 0.01 (95% CI, -0.08 to 0.11) after the inclusion of the multilevel resilience factors. Regarding specific indirect effects, individual-level global resilience (depression symptoms at T3 only) as well as the interpersonal-level relationship reliability and confidence (loneliness at T3 model only) factors were statistically significant. Multilevel resilience factors mediated the bidirectional relationship between loneliness and depression symptoms. Mental health interventions should consider implementing resilience-informed strategies that mitigate depression symptoms and loneliness among older SMM.

**Supplementary Information:**

The online version contains supplementary material available at 10.1007/s10461-024-04416-w.

## Resumen

Los estudios existentes que examinan la resiliencia entre los hombres de minorías sexuales (HSH) se han visto limitados por analizar sólo 1 nivel de resiliencia. Por lo tanto, investigamos el impacto de múltiples niveles de resiliencia en la relación bidireccional entre la soledad y los síntomas de depresión entre los hombres mayores de minorías sexuales. Se recopilaron datos sobre soledad, síntomas de depresión y resiliencia multinivel a lo largo de 3 puntos temporales (octubre de 2016 a marzo de 2017 [T1]; octubre de 2017 a marzo de 2018 [T2]; y octubre de 2018 a marzo de 2019 [T3]) entre 1,264 SMM de 40 años o más que viven con y sin VIH. Se utilizaron modelos de mediación longitudinal para probar el efecto mediador de los factores de resiliencia multinivel en T2 sobre la relación bidireccional entre la soledad y los síntomas de depresión, ajustando por covariables sociodemográficas. Los factores de resiliencia multinivel se asociaron negativamente con la soledad y los síntomas de depresión en T1. El factor de resiliencia global a nivel individual se asoció con menores probabilidades de síntomas de depresión (odds ratio [OR] = 0,50; IC 95%, 0,32 − 0,78), mientras que el factor de confianza en las relaciones a nivel interpersonal (OR = 0.43; 95% CI, 0.24–0.77) y los factores de fiabilidad (OR = 0.36; 95% CI, 0.15–0.84) se asociaron con menores probabilidades de soledad en T3. El efecto total de la soledad en T1 sobre los síntomas depresivos en T3 fue β = 0.20 (95% CI, 0.11–0.28) y se redujo a β = 0.08 (95% CI, -0.04 to 0.20) tras la inclusión de los factores de resiliencia multinivel. El efecto total de los síntomas depresivos en T1 sobre la soledad en T3 fue similar (β = 0.21; 95% CI, 0.13–0.28) con el efecto directo de β = 0.01 (95% CI, -0.08 to 0.11) tras la inclusión de los factores de resiliencia multinivel. En cuanto a los efectos indirectos específicos, los factores de resiliencia global a nivel individual (síntomas de depresión sólo en T3) así como la fiabilidad y confianza de las relaciones interpersonales (soledad en el modelo T3 solamente) fueron estadísticamente significativos. Los factores de resiliencia multinivel mediaron la relación bidireccional entre la soledad y los síntomas de depresión. Las intervenciones de salud mental deberían considerar la implementación de estrategias basadas en la resiliencia que mitiguen los síntomas de la depresión y la soledad entre los SMM mayores.

## Introduction

Sexual minority men (SMM) account for nearly two-thirds of the HIV prevalence in the United States. Now considered a chronic disease, people living with HIV (PLWH) have a longer life expectancy than at the height of the HIV epidemic. The maturing HIV/AIDS epidemic will disproportionately include gay and bisexual men older than age 50 years [1]. Several studies have demonstrated that older SMM living with HIV experience a higher prevalence of loneliness and depression compared with the general population [2–6]. In a recent study, 58% of gay and bisexual men in their mid to late 50s, most of whom were living with HIV, experienced loneliness [7]. Furthermore, those who reported loneliness were more likely to report depression, problem drinking, and tobacco use, and they had fewer relationships. Further evidence highlights that SMM are more likely than non-SMM to face stigma, discrimination, and marginalization, which increases their risk of loneliness and depression symptoms and are exacerbated in the context of HIV [3]. Despite negative psychosocial conditions, many SMM exhibit resilience, which may serve as a protective factor against loneliness and depression symptoms [8].

Resilience is a multifaceted concept that has received growing attention for understanding how individuals promote and protect their health in the presence of adversity [9]. Resilience research has primarily focused on the individual-level processes that nurture a sense of interpersonal efficacy, social self-esteem, mattering, and self-compassion. However, resilience encompasses both assets (i.e., positive factors that reside within the individual) and resources (i.e., positive factors that are external to the individual and help individuals overcome risk) [10, 11]. A recent review of the most current literature on resilience concluded that it is useful to conceptualize resilience as a dynamic and person-situation–defined process, referring to the ability of a person to draw support from available resources (internal and external) when confronted with adversity [12]. In this study, we framed resilience as a multilevel construct. At the individual level, *resilience* refers to qualities—such as personal competence, stress tolerance, acceptance of change, and other positive personal attributes—that enable individuals to thrive in the face of adversity [9–11]. At the interpersonal level, *resilience* includes social support, regarded as an individual’s perception of general support or specific supportive behaviors from people in their social network, and other positive aspects of the social environment (e.g., social cohesion) [8, 11]. *Community-level resilience* refers to communities’ abilities to lean on collective resources and social belonging to promote their health and well-being despite the presence of external stressors [9–11].

Previous studies have demonstrated resilience as a mediator between loneliness, depression, and other health-related outcomes in diverse populations [13–16]. For example, a study with a young adult Danish sample found that an individual’s available resilience resources across an individual-level domain (e.g., perception of self), an interpersonal close relationship domain (e.g., family cohesion), and a community domain (i.e., extra-family social resources) were negatively associated with loneliness, indicating that young adults who had a high degree of resilience also tended to feel less lonely [17]. Another study with a sample of older Italian adults indicated that individuals with greater levels of resilience had a higher level of physical and psychological quality of life satisfaction as measured by depression and anxiety symptoms [18].

To date, there is a dearth of studies that examined the potential role of multilevel resilience in reducing loneliness and depression among older adult SMM with and without HIV. This is critical for a community that has historically faced substantial stigma and discrimination. For example, one study found that older, mostly White, sexual minority adults who reported receiving a lower level of social support had an elevated risk of loneliness [19]. This study, while relevant to older SMM, narrowly focused on one component of resilience (interpersonal level) and did not account for HIV status. The aim of the current study was to examine whether multilevel resilience, operationalized at the individual, interpersonal, and community levels, mediated the bidirectional relationship between depression and loneliness among older SMM living with and without HIV. We hypothesized that there would be a positive bidirectional relationship between depression and loneliness and that the relationship would become weaker with the inclusion of multilevel resilience.

## Methods

### Multicenter AIDS Cohort Study

The Multicenter AIDS Cohort Study (MACS) is a longitudinal study that examined the natural and treated history of HIV among a cohort of SMM in 4 US sites: Baltimore, Maryland/Washington, DC, Chicago, Illinois, Los Angeles, California, and Pittsburgh, Pennsylvania. Beginning in 1984, 7,352 HIV-positive and -negative participants were enrolled over four enrollment waves: 4,954 in 1984–85, 668 in 1987–1991, 1,350 in 2001–2003, and 380 in 2011–2019. Participants completed semiannual clinic visits using audio computer-assisted self-interviews and a standardized clinical examination to collect demographic information, medical history, behavioral assessments, and biospecimens. Details on the MACS study design have been described elsewhere [20, 21].

### Understanding Patterns of Healthy Aging in Men Who Have Sex with Men

Understanding Patterns of Healthy Aging in Men Who Have Sex With Men, a substudy of the MACS, sought to identify and understand psychosocial resilience that promotes healthy aging among middle-aged and older SMM living with and without HIV [22]. It was conducted over 6 MACS visits from April 2016 to March 2019. To be eligible, MACS participants were at least 40 years old on or before April 2016, reported at least 1 incidence of sexual intercourse with another man since enrolling in the MACS, and completed 2 consecutive MACS visits prior to April 2016. A total of 1,317 MACS participants were enrolled in the substudy. The current analyses included 1,264 participants (632 PLWH/632 people living without HIV [PLWOH]) with depression symptoms, loneliness, and resilience data at the following time points: October 2016 to March 2017 (T1), October 2017 to March 2018 (T2), and October 2018 to March 2019 (T3). The median time elapsed between each time point was 6 months.

### Primary Measures

Loneliness was assessed using the UCLA 3-item Loneliness Scale at T1 and T3 [23]. Response values were summed, and scores ranged from 3 to 9 points. Scores less than 6 were categorized as “not lonely” and scores equal to or greater than 6 were categorized as “lonely” [24]. The standardized Cronbach α for this sample was 0.87.

Depression symptoms were assessed using the Center for Epidemiologic Studies Depression (CES-D) Scale at T1 and T3. We calculated the score by summing across all items. A score greater than or equal to 16 indicated the presence of depression symptoms. A score less than 16 was classified as an absence of depression symptoms [25]. The standardized Cronbach α in the sample was 0.82.

### Individual-, Interpersonal-, and Community-Level Resilience

We used the 14-item Resilience Scale to represent *individual-level resilience* [26]. The 7- point Likert scale measured the participants’ attitudes about life and how much they agreed (or disagreed) with 14 statements. Examples of statements included, “My belief in myself gets me through hard times” and “I am determined.” Due to the small sample sizes of some of the responses, the response categories were collapsed into “agree” (strongly agree, agree, and somewhat agree), “neutral” (neutral), and “disagree” (strongly disagree, disagree, and somewhat disagree). “Prefer not to say” responses were treated as missing. The standardized Cronbach α in the sample was 0.96.

For *interpersonal-level resilience*, we used the 9-item Relationship Structures-ECR (Experiences in Close Relationships) Scale to assess attachment patterns of close relationships (avoidance or anxiety) [27]. Examples of statements included “I’m afraid that other people may abandon me” and “I find it easy to depend on others.” The response choices were a 7-point Likert scale ranging from strongly agree to strongly disagree. Negatively framed items were reverse coded so higher values measured positive experiences in close relationships. Due to the size of the responses in some of the categories, the responses were collapsed into “agree” (strongly agree, agree, and somewhat agree), “neutral” (neutral), and “disagree” (strongly disagree, disagree, and somewhat disagree). “Prefer not to say” responses were treated as missing. The standardized Cronbach α was calculated separately for avoidance (0.83) and anxiety (0.91) domains.

The 6-item Psychological Sense of Community Scale represented *community-level resilience* and measured the degree to which members of a community feel that they belong, that they matter to one another and to the group, and that their needs are met by the group [28]. This scale was adapted to the gay male community [29]. Examples of questions included “How much do you feel you can get help from gay men if you need it?” and “How much do you feel like you are a member of the gay male community?” Responses to items 1 through 5 were “none,” “a little,” “some,” “a fair deal,” “a great deal,” and “prefer not to say.” For the analysis, these responses were collapsed into “none/a little,” “some,” and “a fair deal/a great deal.” Possible responses to item 6 were “none,” “a few,” “about half,” “most,” “all,” and “prefer not to say.” These responses were collapsed into “none/a few,” “about half,” and “most/all.” “Prefer not to say” responses were treated as missing. The standardized Cronbach α in the sample was 0.91.

### Covariates

Age was calculated from date of birth and date of visit at T3. Race and ethnicity were categorized as “non-Hispanic White,” “non-Hispanic Black,” “Hispanic,” and “Other.” Education was categorized as “less than a high school diploma,” “high school diploma,” “at least some college,” and “at least some graduate school.” Due to small cell sizes in later enrollment waves, we collapsed the four enrollment waves into two categories (before 2001 and 2001–2019). HIV status (PLWH or PLWOH) was assessed using enzyme-linked immunosorbent assay with a confirmatory Western blot on all participants at their initial visit and every visit for PLWOH at the previous visit. PLWH included all participants who were identified as such at their initial visit and those who seroconverted during study observation. Time since HIV diagnosis is not available for participants. However, since most participants were seropositive at their initial visit, we will use enrollment wave as a proxy for the length of time with HIV.

### Statistical Analysis

Descriptive statistics were generated on the primary measures, resilience scales, and covariates, overall and by HIV status, using medians and interquartile ranges (IQRs) and frequencies and percentages whe6re appropriate. The analysis was conducted in 2 parts: (1) identify psychosocial resilience and (2) determine the effect of these resiliencies on the bidirectional relationship between loneliness and depression.

To identify individual-, interpersonal-, and community-level psychosocial resilience at T2, we used exploratory factor analysis (EFA) and confirmatory factor analysis (CFA) with maximum likelihood estimation. We performed the EFA/CFA in the following steps: (1) randomly divided the sample to create a development data set for the EFA (*n* = 632) and a data set to test using CFA (*n* = 632); (2) performed EFA at each resilience level scale to explore the number of potential latent factors using model fit criteria then confirmed the factors through CFA; and (3) combined all factors across resilience levels and confirmed latent factors structure of the final measurement model using CFA in the full data set. Latent factor identification and retention were based on the following model fit criteria and theoretical construct of the following factors: Kaiser criterion (retention of factors with eigenvalues > 1), factor loadings ≥ |0.4|, root mean square error of approximation (RMSEA) < 0.05 (including its 95% CI), comparative fit index (CFI) > 0.95, Tucker-Lewis Index (TLI) > 0.95, and the standardized root mean square residual (SRMR) < 0.1 [30–32].

**Fig. 1:**
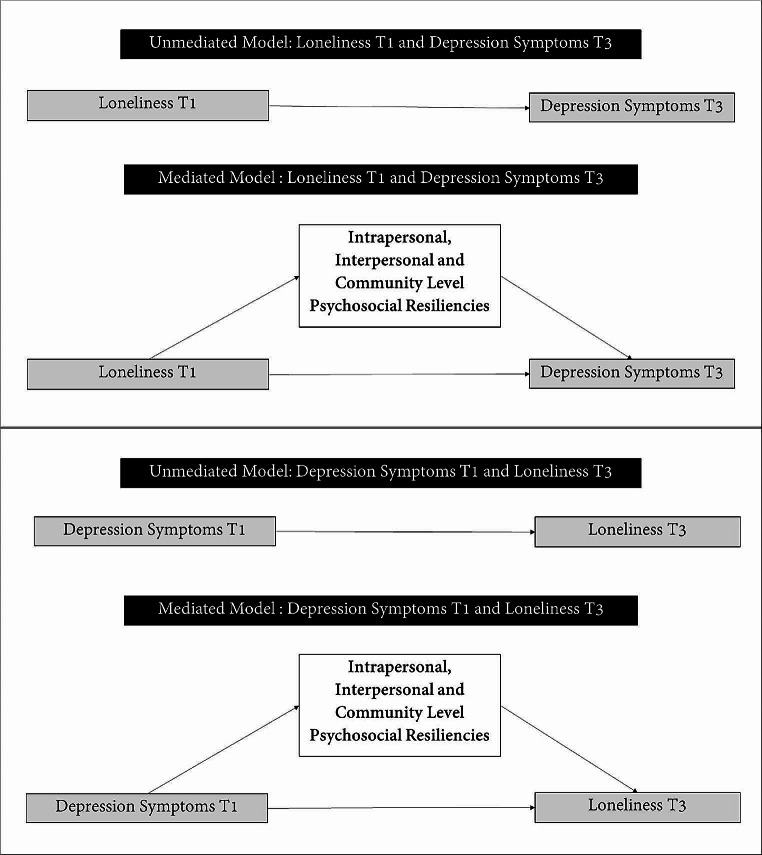
Diagram of unmediated and mediated models for both depression symptoms (Panel **A**) and loneliness (Panel **B**) as outcomes

To explore the bidirectional relationship between loneliness and depressive symptoms, we examined the effect of the resilience factors in 2 mediation models: (1) loneliness at T1 as the independent variable and depression symptoms at T3 as the outcome variable (Fig. 1 panel A) and (2) depression symptoms at T1 as the independent variable and loneliness at T3 as the outcome variable (Fig. 1 panel B) [33]. We adjusted for covariates (age, HIV status, enrollment wave and education) in the mediation model. We included an interaction term between HIV status and enrollment wave to discern in differences in the relationship between HIV status and loneliness/depression symptoms outcome by enrollment wave.

Model fit statistics (RMSEA, CFI/TLI, SRMR) and standardized factor loadings were reported for the CFA model. Odds ratios (ORs), and total direct and indirect effects along with their 95% CIs were reported. We used the Sobel test to evaluate the significance of the medication effect. Statistical significance was set at *p* < 0.05. Analyses were performed in Mplus version 8.7 and SAS version 9.4.

### Missing Data

In the EFA/CFA, missing values are handled by full information maximum likelihood estimation [34], meaning that all available data are used. However, if a participant is missing all data, they would be dropped from the analysis. Of 1,264 participants in the sample, 1 participant was removed for missing data points on every item of the scales. In the mediation model, an additional 210 participants were removed for missing independent variables or covariates. A χ^2^ test was performed on missingness of loneliness and depression symptoms and the remaining variables to reveal whether it was related to other variable values in the data. We did not find any significant relationship of missingness on loneliness and depression symptoms among the variables. The final analytic sample included 1,054 SMM.

## Results

### Descriptive Statistics

The median age of participants was 61 years (IQR, 55–67). Most of the participants were non-Hispanic White (69%), had at least some college education (85.3%) and were enrolled before 2001 (63%). At T1, depression symptoms and loneliness were reported in 18.3% and 28.1% of the participants, respectively. At T3, depression symptoms were reported in 19.3% of the participants, while loneliness was reported in 27.6%. Among PLWH, the median age was 58 years (IQR, 53–64), 57.6% were non-Hispanic White, 81.2% had at least some college education and 50.6% were enrolled before 2001. Depression symptoms were reported in 22.6% and 22.8% of PLWH at T1 and T3, respectively. The prevalence of loneliness among PLWH was 30.5% and 29.4% at T1 and T3, respectively. Among PLWOH, the median age was 63 years (IQR, 57–69), 80.4% were non-Hispanic White, 89.6% had at least some college education and 75.3% were enrolled before 2001. Depression symptoms were reported among PLWOH at 13.9% and 15.8% at T1 and T3, respectively. Loneliness prevalence among PLWOH was 25.6% and 25.8% at T1 and T3, respectively (Table 1). Descriptive statistics for each scale were reported by HIV status in Supplemental Table 1.

**Table 1:** Analytic sample characteristics overall and by HIV status

	PLWOH (*n* = 632)	PLWH (*n* = 632)	All participants (*n* = 1,264)
**Age distribution, median (IQR), y**	63 (57–69)	58 (53–64)	61 (55–67)
**Race and ethnicity, n (%)**			
Non-Hispanic White	508 (80.4)	364 (57.6)	872 (69.0)
Non-Hispanic Black	80 (12.7)	176 (27.9)	256 (20.3)
Hispanic	32 (5.1)	80 (12.7)	112 (8,9)
Other	12 (1.9)	12 (1.9)	24 (1.9)
**Education, n (%)**			
Less than high school	14 (2.2)	33 (5.2)	47 (3.7)
High school	52 (8.2)	86 (13.6)	138 (10.9)
College	379 (60.0)	400 (63.3)	779 (61.6)
Graduate	187 (29.6)	113 (17.9)	300 (23.7)
**Enrollment Wave**			
Before 2001	476 (75.3)	320 (50.6)	796 (63.0)
2001–2019	156 (24.7)	312 (49.4)	468 (37.0)
**Depression symptoms at time 1, n (%)**			
None	432 (68.4)	369 (58.4)	801 (63.4)
Depression symptoms	88 (13.9)	143 (22.6)	231 (18.3)
Missing	112 (17.7)	120 (19.0)	232 (18.4)
**Depression symptoms at time 3, n (%)**			
None	412 (65.2)	375 (59.3)	787 (62.3)
Depression symptoms	100 (15.8)	144 (22.8)	244 (19.3)
Missing	120 (19.0)	113 (17.9)	233 (18.4)
**Loneliness at time 1, n (%)**			
Not lonely	340 (53.8)	286 (45.3)	626 (49.5)
Lonely	162 (25.6)	193 (30.5)	355 (28.1)
Missing	130 (20.6)	153 (24.2)	283 (22.4)
**Loneliness at time 3, n (%)**			
Not lonely	332 (52.5)	297 (47.0)	629 (49.8)
Lonely	163 (25.8)	186 (29.4)	349 (27.6)
Missing	137 (21.7)	149 (23.6)	286 (22.6)

PLWH: people living with HIV; PLWOH: people living without HIV

### Exploratory Factor Analysis

For the 14-item resilience scale (individual-level resilience), there was 1 factor with an eigenvalue greater than 1. The 1-factor model represented the best fitting model and the model fit statistics were as follows: (1) RMSEA of 0.124 (90% CI, 0.118–0.129), (2) CFI/TLI of 0.995/0.994, and (3) SRMR of 0.034. For the 9-item Relationship Structures-ECR Scale (interpersonal level), 3 factors had an eigenvalue greater than 1. However, the 4-factor model had more favorable model fit statistics vs. the three-factor model. The fit statistics for the 4-factor model were as follows: (1) RMSEA of 0.000 (90% CI, 0.000-0.034), (2) CFI/TLI of 1.000/1.000, and (3) SRMR of 0.010. For the 6-item Psychological Sense of Community Scale (community level), 1 factor had an eigenvalue greater than 1. However, the 2-factor model had better model fit statistics and maintained the factor structure published in previous literature [12]: (1) RMSEA of 0.030 (90% CI, 0.000-0.063), (2) CFI/TLI of 1.000/1.000, and (3) SRMR of 0.009. Factor loadings are reported in Table 2.

**Table 2:** Geomin rotated loadings for the best model fit for each multilevel resilience

14-Item Resilience Scale	Factor 1			
My belief in myself gets me through hard times.	0.787 (z = 80.07) *			
I am determined.	0.978 (z = 234.46) *			
I can get through difficult times because I’ve experienced difficulty before.	0.954 (z = 295.66) *			
I have self-discipline.	0.960 (z = 282.30) *			
When I’m in a difficult situation, I can usually find my way out of it.	0.928 (z = 220.78) *			
I am friends with myself.	0.953 (z = 362.64) *			
I feel that I can handle many things at a time.	0.945 (z = 265.16) *			
I keep interested in things.	0.935 (z = 222.36) *			
I can usually find something to laugh about.	0.945 (z = 283.69) *			
I usually manage o8ne way or another.	0.961 (z = 283.83) *			
My life has meaning.	0.954 (z = 294.75) *			
I feel proud that I have accomplished things in life.	0.936 (z = 273.18) *			
In an emergency, I’m someone people can generally rely on.	0.933 (z = 252.91) *			
I usually take things in stride.	0.787 (z = 80.07) *			
***9-Item Relationship Structures-ECR***	**Factor 1**	**Factor 2**	**Factor 3**	**Factor 4**
I’m afraid that other people may abandon me. (R)	0.826 (z = 8.41) *	0.015 (z = 0.29)	0.028 (z = 0.51)	-0.036 (z= -1.05)
I feel comfortable opening up to others. (R)	0.798 (z = 6.30) *	0.001 (z = 0.04)	-0.005 (z= -0.14)	0.123 (z = 0.97)
I find it easy to depend on others.	0.184 (z = 1.58)	0.155 (z = 0.88)	-0.019 (z= -0.77)	0.732 (z = 2.93) *
I usually discuss my problems and concerns with others.	-0.013 (z= -0.46)	0.969 (z = 8.30) *	-0.003 (z= -0.08)	-0.032 (z= -2.47) *
I worry that others won’t care about me as much as I care about them. (R)	0.023 (z = 0.58)	0.11 (z = 1.77)	0.664 (z = 11.26) *	0.077 (z = 1.26)
I prefer to show others how I feel deep down. (R)	-0.057 (z= -1.32)	0.044 (z = 1.61)	0.956 (z = 14.20) *	0.000 (z = 0.01)
It helps to turn to people in times of need.	-0.041 (z= -1.56)	-0.041 (z= -1.32)	0.03 (z = 1.01)	1.049 (z = 6.81) *
I talk things over with people.	0.018 (z = 0.57)	0.760 (z = 4.78) *	0.044 (z = 0.71)	0.084 (z = 0.73)
I often worry that other people do not really care for me. (R)	0.231 (z = 3.13) *	-0.104 (z= -1.35)	0.708* (z = 8.82)	-0.025 (z= -0.55)
***Community-Level Resilience***	**Factor 1**	**Factor 2**		
How much do you feel like you belong in the gay male community?	0.877* (z = 18.05)	0.097 (z = 1.82)		
How much do you feel that you can get help from gay men if you need it?	0.209 (z = 0.76)	0.655* (z = 2.39)		
How much do you feel that you help other people in the gay male community when they need help?	-0.001 (z= -0.20)	0.872* (z = 17.13)		
How much do you feel that you are a member of the gay male community?	1.072* (z = 19.79)	-0.097 (z= -1.67)		
How many of your needs do you feel are met by the gay male community?	0.693* (z = 6.53)	0.129 (z = 1.14)		
How much do you feel a part of the gay male community?	0.980* (z = 200.85)	0.002 (z = 0.62)		

*p < 0.05; Bolded values indicate the highest loading on the respective factor

### Confirmatory Factor Analysis

We confirm the following factors identified by the EFA at their respective level using the CFA test data set: (1) individual level (1 factor: global resilience); (2) interpersonal level (4 factors: relationship closeness/relationship reliability/relationship confidence/relationship openness); and (3) community level (2 factors: community belonging/community helping). All of the factors were included in a single measurement model. The model fit indices of the final measurement model were as follows: (1) RSMEA of 0.040 (90% CI, 0.037–0.043); (2) CFI/TLI of 0.990/0.989; and (3) SRMR of 0.056, indicating good model fit. Items from each scale loaded strongly on their respective factors (Table 3). All resilience factors were negatively associated with depression symptoms and loneliness at T1. Further details are reported in Supplemental Table 2. The correlation coefficients between loneliness, depression symptoms, and the resilience factors are reported in Table 4.

**Table 3:** Factor loadings of the multilevel resilience factors^a^

Individual-Level Resilience	Factor loading
**Global resilience**	
My belief in myself gets me through hard times.	0.916 (z = 76.97) ***
I am determined.	0.912 (z = 69.87) ***
I can get through difficult times because I’ve experienced difficulty before.	0.881 (z = 50.79) ***
I have self-discipline.	0.763 (z = 31.82) ***
When I’m in a difficult situation, I can usually find my way out of it.	0.948 (z = 74.42) ***
I am friends with myself.	0.913 (z = 67.87) ***
I feel that I can handle many things at a time.	0.849 (z = 46.95) ***
I keep interested in things.	0.882 (z = 52.52) ***
I can usually find something to laugh about.	0.872 (z = 48.46) ***
I usually manage one way or another.	0.878 (z = 40.60) ***
My life has meaning.	0.891 (z = 57.55) ***
I feel proud that I have accomplished things in life.	0.907 (z = 60.85) ***
In an emergency, I’m someone people can generally rely on.	0.834 (z = 36.15) ***
I usually take things in stride.	0.857 (z = 49.49) ***
***2Interpersonal-Level Resilience***	
**Relationship closeness**	
I’m afraid that other people may abandon me. (R)	0.774 (z = 27.59) ***
I feel comfortable opening up to others. (R)	0.620 (z = 20.28) ***
**Relationship reliability**	
I find it easy to depend on others.	0.731 (z = 16.48) ***
It helps to turn to people in times of need.	0.722 (z = 14.94) ***
**Relationship confidence**	
I worry that others won’t care about me as much as I care about them. (R)	0.845 (z = 56.36) ***
I prefer to show others how I feel deep down. (R)	0.741 (z = 29.33) ***
I often worry that other people do not really care for me. (R)	0.874 (z = 62.85) ***
**Relationship openness**	
I usually discuss my problems and concerns with others.	0.947 (z = 47.20) ***
I talk things over with people.	0.974 (z = 48.16) ***
***Community-Level Resilience***	
**Community belonging**	
How much do you feel like you belong in the gay male community?	0.988 (z = 169.89) ***
How much do you feel that you are a member of the gay male community?	0.980 (z = 164.91) ***
How many of your needs do you feel are met by the gay male community?	0.794 (z = 35.12) ***
How much do you feel a part of the gay male community?	0.971 (z = 153.41) ***
**Community helping**	
How much do you feel that you can get help from gay men if you need it?	0.920 (z = 68.81) ***
How much do you feel that you help other people in the gay male community when they need help?	0.868 (z = 57.58) ***

(R), item values reversed coded. *p < 0.05; **p < 0.01; ***p < 0.001

^a^Model fit: root mean square error of approximation = 0.040 (95% CI, 0.037–0.043); comparative fit index/Tucker-Lewis Index = 0.990/0.989; and standardized root mean square residual = 0.056

**Table 4:** Correlation coefficients for loneliness, depression symptoms, and resilience factors

Individual-Level Resilience	Loneliness at time 1	Depression at time 1	Loneliness at time 3	Depression at time 3
Global resilience	-0.417	-0.581	-0.391	-0.490
***Interpersonal-Level Resilience***				
Relationship closeness	-0.598	-0.580	-0.495	-0.431
Relationship reliability	-0.498	-0.490	-0.412	-0.308
Relationship confidence	-0.560	-0.483	-0.508	-0.389
Relationship openness	-0.413	-0.313	-0.357	-0.277
***Community-Level Resilience***				
Community belonging	-0.375	-0.241	-0.373	-0.246
Community helping	-0.413	-0.336	-0.356	-0.255

### Bidirectional Adjusted Association Between Depression Symptoms and Loneliness with and without Multilevel Resilience

Prior to the introduction of the multilevel resilience factors into models, participants reporting depression symptoms at T1 had greater odds of loneliness at T3 (OR = 2.46; 95% CI, 1.62–3.72). Similarly, participants reporting loneliness at T1 had greater odds of depression symptoms at T3 (OR = 2.33; 95% CI, 1.55–3.50). After the introduction of the multilevel resilience factors as mediators at T2, the relationship between loneliness (T1) and depression symptoms (T3) (OR = 1.45; 95% CI, 0.83–2.54) and between depression symptoms (T1) and loneliness (T3) (OR = 1.09; 95% CI, 0.62–1.91) were no longer statistically significant.

For the association between the multilevel resilience factors and depression symptoms at T3, the global resilience factor (OR = 0.50; 95% CI, 0.32–0.79) was associated with lower odds of depression symptoms. In examining the relationship between multilevel resilience factors and loneliness at T3, relationship reliability (OR = 0.41; 95% CI: 0.19–0.86) and confidence (OR = 0.42; 95% CI, 0.23–0.77) factors had lower odds of loneliness (Table 5).

**Table 5:** Adjusted logistic model results for depression and loneliness outcomes

	Depression Symptoms Outcome	Loneliness Outcome
Individual-Level Resilience	OR (95% CI)	OR (95% CI)
Global resilience^a^	0.50 (95% CI: 0.32–0.78) **	0.77 (95% CI: 0.41–1.47)
***Interpersonal-Level Resilience***		
Relationship closeness^a^	0.89 (95% CI: 0.56–1.42)	0.94 (95% CI: 0.60–1.48)
Relationship reliability^a^	0.77 (95% CI: 0.28–2.13)	0.36 (95% CI: 0.15–0.84) *
Relationship confidence^a^	0.76 (95% CI: 0.43–1.34)	0.43 (95% CI: 0.24–0.77) ***
Relationship openness^a^	1.00 (95% CI: 0.64–1.58)	1.08 (95% CI: 0.65–1.79)
***Community-Level Resilience***		
Community belonging^a^	0.79 (95% CI: 0.53–1.17)	0.69 (95% CI: 0.47–1.01)
Community helping^a^	1.12 (95% CI: 0.72–1.74)	1.06 (95% CI: 0.72–1.58)
***Covariates***		
Loneliness at T1 (vs. No Loneliness)	1.42 (95% CI: 0.78–2.59)	12.20 (95% CI: 7.80–19.09) ***
Depressive Symptoms at T1 (vs. No Depressive Symptoms)	9.97 (95% CI: 6.41–15.52) ***	1.00 (95% CI: 0.55–1.81)
Age (per year increase)	1.00 (95% CI: 0.97–1.04)	0.98 (95%CI: 0.94–1.01)
PLWH (vs. PLWOH)	0.99 (95% CI: 0.65–1.50)	0.72 (95%CI: 0.47–1.10)
Black Non-Hispanic (vs. White Non-Hispanic)	0.61 (95% CI: 0.33–1.13)	0.94 (95% CI: 0.54–1.64)
Hispanic (vs. White Non-Hispanic)	1.20 (95% CI: 0.61–2.37)	0.60 (95% CI: 0.25–1.41)
Other (vs. White Non-Hispanic)	0.52 (95% CI: 0.16–1.71)	0.16 (95% CI: 0.03–0.88) *
Less than High School (vs. Graduate School)	1.34 (95% CI: 0.43–4.15)	0.79 (95% CI: 0.26–2.40)
High School (vs. Graduate School)	2.74 (95% CI: 1.16–6.40) *	0.75 (95% CI: 0.36–1.57)
College (vs. Graduate School)	2.08 (95% CI: 1.16–3.73) *	0.97 (95% CI: 0.59–1.62)
After 2001 Enrollment (vs. Before 2001 Enrollment)	1.55 (95% CI: 0.90–2.68)	1.31 (95% CI: 0.77–2.25)

*p < 0.05; **p < 0.01; ***p < 0.001; OR: Odds Ratio; CI: Confidence Interval; ^a^ per unit increase in factor score

Regarding covariates in the adjusted model, depression symptoms at T1 (vs. no depressive symptoms; OR = 9.91; 95% CI: 6.40-15.36) and the attainment of high school (OR = 2.68; 95% CI: 1.18–6.05) or college (OR = 2.05; 95% CI:1.16–3.62) education (vs. graduate education) were associated with increased odds of depression symptoms at T3. Loneliness at T1 (OR = 12.20; 95% CI: 7.80-19.09), other race (OR = 0.17; 95% CI: 0.03–0.86), and enrollment after 2001 (vs. before 2001; OR = 1.63; 95% CI: 1.03–2.60) were associated with increased odds of loneliness at T3. HIV status was not shown to be statistically associated with loneliness or depression symptoms at T3. The interaction term between HIV status and enrollment wave were not statistically significant.

### Bidirectional Mediation Model Results

**Fig. 2:**
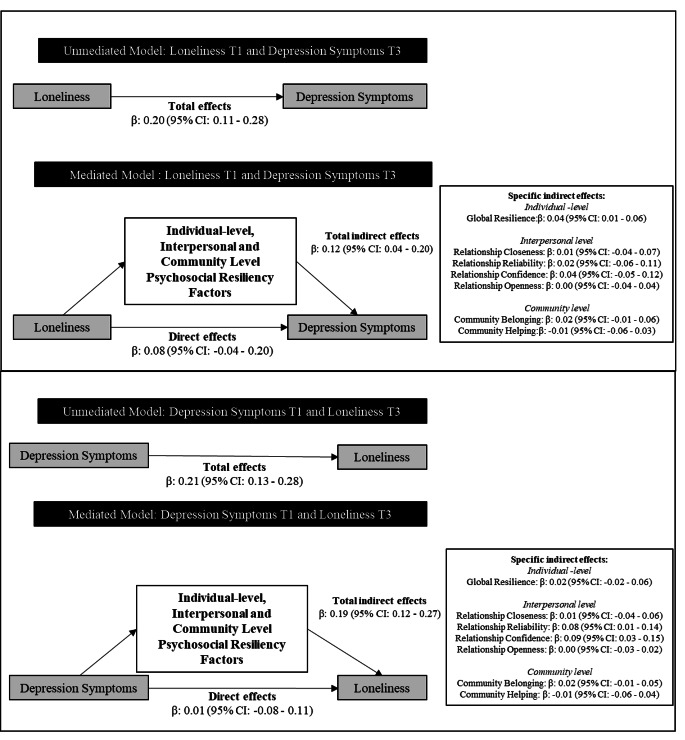
Total direct and total indirect effects of the adjusted bidirectional mediation analysis of depression symptoms and loneliness

The total effect of loneliness at T1 on depressive symptoms at T3 was β of 0.20 (95% CI, 0.11–0.28). The direct effect was reduced to β of 0.08 (95% CI, -0.04–0.20) and was no longer statistically significant after the inclusion of the multilevel resilience factors (Fig. 2). The total indirect effects of the multilevel resilience factors were β of 0.12 (95% CI, 0.04–0.20). Among the mediating multilevel resilience factors, only the global resilience (β = 0.04; 95% CI, 0.01–0.06; Sobel test statistic = 2.72; *p* = 0.006) factor had statistical significant indirect effects (Fig. 2).

The total effect of depressive symptoms at T1 on loneliness at T3 was similar (β = 0.20; 95% CI, 0.13–0.28) with the direct effect of β of 0.01 (95% CI, -0.08–0.11) after the inclusion of the multilevel resilience factors (Fig. 2). The total indirect effect of the multilevel resilience factors was β of 0.19 (95% CI, 0.12–1.41). The relationship reliability (β = 0.08; 95% CI, 0.01–0.14; Sobel test statistic = 2.21; *p* = 0.03) and confidence (β = 0.09; 95% CI, 0.03–0.15; Sobel test statistic = 2.71; *p* = 0.007), factors had statistically significant indirect effects (Fig. 2).

## Discussion

The concept of multilevel resilience provides an important lens through which to examine the psychosocial health and well-being of older SMM living with and without HIV. To our knowledge, this study was the first to examine the impact of multiple levels of resilience on the bidirectional relationship between loneliness and depression symptoms among older SMM living with and without HIV.

The results of our study demonstrated that multilevel resilience disrupted the path between loneliness and depression symptoms in this population. These findings corroborated prior studies that examined the relationships between mental health–related outcomes, such as loneliness and depression symptoms, and resilience factors [13–18]. Our study added to the literature by elucidating how distinct levels of resilience affect the bidirectional relationship between loneliness and depression differentially. Specifically, individual-level resilience alone mediated depression symptoms at T3, while all 3 levels of resilience—the individual, interpersonal (i.e., relationship confidence), and community (i.e., community belonging) levels—served as protective factors against loneliness at T3. Previous studies have demonstrated that older SMM are likely to experience situations of adversity, what is referred to in the literature as “minority stressors” [18] such as homophobic discrimination, during their lifetime, which is associated with having significant depression symptoms.

Consistent with previous studies [18, 19], the study results revealed that strong individual-level resilience in the face of adversities mitigated older SMM’s disproportionate risk of depression. One plausible explanation for these results was that older SMM who display robust individual-level resilience may perceive sexual identity discrimination not as a threat to their health and well-being, but as an opportunity to overcome a challenge (e.g., “When I’m in a difficult situation, I can usually find my way out of it”). These adverse life experiences for some older SMM may build their resilience levels and adaptive abilities, which, in turn, may help them cope with contexts of risk such as discrimination. It is important to note that older SMM came of age when there were limited legal protections for LGBTQ + communities [18], and these limited protections likely facilitated more individual-level resilience among this population.

Further, the study results illuminated that multilevel resilience mediated the outcome of loneliness for older SMM at T3. In other words, individual-level resilience converged with a higher level of relationship confidence, demonstrating a sense of security that emerged through older SMM’s interpersonal relationships (e.g., “I do not worry that others won’t care about me as much as I care about them”) as well as a higher sense of community belonging (i.e., a deeper connection with and support from the gay male community).

A feasible interpretation for these results was that a multifactorial model of resilience mediated the negativ2e impact of loneliness among older SMM. This model of resilience is in line with the conceptualization of resilience as a process in which individual and environmental factors interact [10–12]. Specifically, components of the self, relationships with others, and community support mediated the adverse experience of loneliness among older SMM. These results shed light on the importance of disentangling the different levels of resilience to understand their impact on loneliness among older SMM.

### Limitations and Strengths of the Study

The study had some limitations. First, although the sample size was suitable, we used convenience sampling for our recruitment design, and our sample consisted of mostly non-Hispanic White SMM, thereby reducing the generalizability of our findings. Our study design should, therefore, be replicated with larger, alternative community samples of ethnically and racially diverse older SMM to understand the impact of multilevel resilience across a heterogeneous group of SMM. Future studies are needed to explore any differences across the individual, interpersonal, and community levels of resilience between White older SMM and those of other races and ethnicities. Second, future waves of longitudinal data will allow us to investigate how multilevel resilience changes over time in older SMM. Future surveillance of resilience in mental health contexts for SMM may also benefit from community input.

Notwithstanding these limitations, the study also had several strengths. First, rather than examining 1 specific level of resilience, this study was innovative in investigating 3 levels of resilience (i.e., individual, interpersonal, and community levels) and potential implications for intervention for older SMM with and without HIV. Second, the longitudinal data allowed us to make causal inferences related to the multilevel resilience factors mediating the bidirectional relationship between loneliness and depression symptoms. Third, our study also included older SMM without HIV.

### Implications for Practice

Models of successful aging highlight the importance of identifying factors that are modifiable through interventions to promote the health and well-being of older adults living with and without HIV. The current study shed light on the theoretical and practical significance of multilevel resilience to inform the development of interventions to mediate against loneliness and depression symptoms among older SMM living with and without HIV. To mitigate the minority stressors that older SMM are likely to experience (e.g., stigma, discrimination, and marginalization) and decrease their risk of loneliness and depression symptoms, it is important to promote interventions that not only build their individual-level resilience, but also increase opportunities to strengthen positive interpersonal relationships and community support.

## Conclusions

The results of this study provide useful information for the development of mental health, interpersonal, and community-level interventions that implement resilience-informed strategies and strengths-based approaches for older SMM. Leveraging multilevel resilience can disrupt the path between loneliness and depression symptoms in this population.

## Electronic Supplementary Material

Below is the link to the electronic supplementary material.

Supplementary Material 1
