# Supplementary material for: Disrupting the Path from Depression to Loneliness: Multilevel Resilience among Older Sexual Minority Men with and without HIV
Source: AIDS Behav. 2024 Jul 24;28(11):3574–86. doi: 10.1007/s10461-024-04416-w (PMC11471709; doi:10.1007/s10461-024-04416-w)
Supplement: Supplementary file 1 — Supplementary Material 1 [file 10461_2024_4416_MOESM1_ESM.docx]

**Supplemental Table 1. Descriptive Statistics of the Scales That Compose Multilevel Resilience Overall and by HIV Status**

|  | **HIV negative**  **(n = 632)** | **HIV positive**  **(n = 632)** | **All participants**  **(n = 1,264)** |
| --- | --- | --- | --- |
| ***Individual-level resilience*** | | | |
| **My belief in myself gets me through hard times, n (%)** | | | |
| Disagree | 27 (4.3) | 35 (5.5) | 62 (4.9) |
| Neutral | 46 (7.3) | 63 (10.0) | 109 (8.6) |
| Agree | 434 (68.7) | 384 (60.8) | 818 (64.7) |
| Missing | 125 (19.8) | 150 (23.7) | 275 (21.8) |
| **I am determined, n (%)** | | | |
| Disagree | 24 (3.8) | 31 (4.9) | 55 (4.4) |
| Neutral | 41 (6.5) | 54 (8.5) | 95 (7.5) |
| Agree | 442 (69.9) | 395 (62.5) | 837 (66.2) |
| Missing | 125 (19.8) | 152 (24.1) | 277 (21.9) |
| **I can get through difficult times because I've experienced difficulty before, n (%)** | | | |
| Disagree | 17 (2.7) | 28 (4.4) | 45 (3.6) |
| Neutral | 35 (5.5) | 44 (7.0) | 79 (6.3) |
| Agree | 453 (71.7) | 411 (65.0) | 864 (68.4) |
| Missing | 127 (20.1) | 149 (23.6) | 276 (21.8) |
| **I have self-discipline, n (%)** | | | |
| Disagree | 49 (7.8) | 61 (9.7) | 110 (8.7) |
| Neutral | 51 (8.1) | 58 (9.2) | 109 (8.6) |
| Agree | 406 (64.2) | 363 (57.4) | 769 (60.8) |
| Missing | 126 (19.9) | 150 (23.7) | 276 (21.8) |
| **When I'm in a difficult situation, I can usually find my way out of it, n (%)** | | | |
| Disagree | 12 (1.9) | 22 (3.5) | 34 (2.7) |
| Neutral | 25 (4.0) | 39 (6.2) | 64 (5.1) |
| Agree | 465 (73.6) | 421 (66.6) | 886 (70.1) |
| Missing | 130 (20.6) | 150 (23.7) | 280 (22.2) |
| **I am friends with myself, n (%)** | | | |
| Disagree | 26 (4.1) | 37 (5.9) | 63 (5.0) |
| Neutral | 42 (6.7) | 46 (7.3) | 88 (7.0) |
| Agree | 438 (69.3) | 398 (63.0) | 836 (66.1) |
| Missing | 126 (19.9) | 151 (23.9) | 277 (21.9) |
| **I feel that I can handle many things at a time, n (%)** | | | |
| Disagree | 49 (7.8) | 57 (9.0) | 106 (8.4) |
| Neutral | 43 (6.8) | 46 (7.3) | 89 (7.0) |
| Agree | 417 (66.0) | 377 (59.7) | 794 (62.8) |
| Missing | 123 (19.5) | 152 (24.1) | 275 (21.8) |
| **I keep interested in things, n (%)** | | | |
| Disagree | 19 (3.0) | 40 (6.3) | 59 (4.7) |
| Neutral | 35 (5.5) | 46 (7.3) | 81 (6.4) |
| Agree | 454 (71.8) | 391 (61.9) | 845 (66.9) |
| Missing | 124 (19.6) | 155 (24.5) | 279 (22.1) |
| **I can usually find something - laugh about, n (%)** | | | |
| Disagree | 24 (3.8) | 36 (5.7) | 60 (4.7) |
| Neutral | 36 (5.7) | 48 (7.6) | 84 (6.6) |
| Agree | 448 (70.9) | 399 (63.1) | 847 (67.0) |
| Missing | 124 (19.6) | 149 (23.6) | 273 (21.6) |
| **I usually manage one way or another, n (%)** | | | |
| Disagree | 8 (1.3) | 19 (3.0) | 27 (2.1) |
| Neutral | 30 (4.8) | 39 (6.2) | 69 (5.5) |
| Agree | 469 (74.2) | 425 (67.3) | 894 (70.7) |
| Missing | 125 (19.8) | 149 (23.6) | 274 (21.7) |
| **My life has meaning, n (%)** | | | |
| Disagree | 23 (3.6) | 36 (5.7) | 59 (4.7) |
| Neutral | 39 (6.2) | 50 (7.9) | 89 (7.0) |
| Agree | 441 (69.8) | 396 (62.7) | 837 (66.2) |
| Missing | 129 (20.4) | 150 (23.7) | 279 (22.1) |
| **I feel proud that I have accomplished things in life, n (%)** | | | |
| Disagree | 19 (3.0) | 27 (4.3) | 46 (3.6) |
| Neutral | 32 (5.1) | 43 (6.8) | 75 (5.9) |
| Agree | 454 (71.8) | 410 (64.9) | 864 (68.4) |
| Missing | 127 (20.1) | 152 (24.1) | 279 (22.1) |
| **In an emergency, I'm someone people can generally rely on, n (%)** | | | |
| Disagree | 18 (2.9) | 25 (4.0) | 43 (3.4) |
| Neutral | 30 (4.8) | 49 (7.8) | 79 (6.3) |
| Agree | 459 (72.6) | 408 (64.6) | 867 (68.6) |
| Missing | 125 (19.8) | 150 (23.7) | 275 (21.8) |
| **I usually take things in stride, n (%)** | | | |
| Disagree | 30 (4.8) | 46 (7.3) | 76 (6.0) |
| Neutral | 47 (7.4) | 70 (11.1) | 117 (9.3) |
| Agree | 425 (67.3) | 365 (57.8) | 790 (62.5) |
| Missing | 130 (20.6) | 151 (23.9) | 281 (22.2) |
| ***Interpersonal-level resilience*** | | | |
| **I'm afraid that other people may abandon me, n (%)** | | | |
| Disagree | 335 (53.0) | 297 (47.0) | 632 (50.0) |
| Neutral | 146 (11.6) | 146 (11.6) | 146 (11.6) |
| Agree | 87 (13.8) | 99 (15.7) | 186 (14.7) |
| Missing | 141 (22.3) | 159 (25.2) | 300 (23.7) |
| **I don't feel comfortable opening up - others, n (%)** | | | |
| Disagree | 265 (41.9) | 221 (35.0) | 486 (38.4) |
| Neutral | 74 (11.7) | 82 (13.0) | 156 (12.3) |
| Agree | 158 (25.0) | 169 (26.7) | 327 (25.9) |
| Missing | 135 (21.4) | 160 (25.3) | 295 (23.3) |
| **I find it easy - depend on others, n (%)** | | | |
| Disagree | 156 (24.7) | 155 (24.5) | 311 (24.6) |
| Neutral | 70 (11.1) | 79 (12.5) | 149 (11.8) |
| Agree | 270 (42.7) | 233 (36.9) | 503 (39.8) |
| Missing | 136 (21.5) | 165 (26.1) | 301 (23.8) |
| **I usually discuss my problems and concerns with others, n (%)** | | | |
| Disagree | 62 (9.8) | 80 (12.7) | 142 (11.2) |
| Neutral | 51 (8.1) | 55 (8.7) | 106 (8.4) |
| Agree | 382 (60.4) | 338 (53.5) | 720 (57.0) |
| Missing | 137 (21.7) | 159 (25.2) | 296 (23.4) |
| **I worry that others won't care about me as much as I care about them, n (%)** | | | |
| Disagree | 287 (45.4) | 276 (43.7) | 563 (44.5) |
| Neutral | 82 (13.0) | 83 (13.1) | 165 (13.1) |
| Agree | 119 (18.8) | 114 (18.0) | 233 (18.4) |
| Missing | 144 (22.8) | 159 (25.2) | 303 (24.0) |
| **I prefer not - show others how I feel deep down, n (%)** | | | |
| Disagree | 247 (39.1) | 197 (31.2) | 444 (35.1) |
| Neutral | 73 (11.6) | 83 (13.1) | 156 (12.3) |
| Agree | 176 (27.9) | 193 (30.5) | 369 (29.2) |
| Missing | 136 (21.5) | 159 (25.2) | 295 (23.3) |
| **It helps - turn - people in times of need, n (%)** | | | |
| Disagree | 12 (1.9) | 21 (3.3) | 33 (2.6) |
| Neutral | 32 (5.1) | 42 (6.7) | 74 (5.9) |
| Agree | 449 (71.0) | 413 (65.4) | 862 (68.2) |
| Missing | 139 (22.0) | 156 (24.7) | 295 (23.3) |
| **I talk things over with people, n (%)** | | | |
| Disagree | 52 (8.2) | 47 (7.4) | 99 (7.8) |
| Neutral | 35 (5.5) | 41 (6.5) | 76 (6.0) |
| Agree | 409 (64.7) | 386 (61.1) | 795 (62.9) |
| Missing | 136 (21.5) | 158 (25.0) | 294 (23.3) |
| **I often worry that other people do not really care for me, n (%)** | | | |
| Disagree | 319 (50.5) | 280 (44.3) | 599 (47.4) |
| Neutral | 77 (12.2) | 90 (14.2) | 167 (13.2) |
| Agree | 95 (15.0) | 104 (16.5) | 199 (15.7) |
| Missing | 141 (22.3) | 158 (25.0) | 299 (23.7) |
| ***Community-Level Resilience*** | | | |
| **How much do you feel like you belong in the gay male community?, n (%)** | | | |
| None - A little | 124 (19.6) | 133 (21.0) | 257 (20.3) |
| Some | 112 (17.7) | 112 (17.7) | 224 (17.7) |
| A fair amount - a great deal | 253 (40.0) | 220 (34.8) | 473 (37.4) |
| Missing | 143 (22.6) | 167 (26.4) | 310 (24.5) |
| **How much do you feel that you can get help from gay men if you need it?, n (%)** | | | |
| None - A little | 117 (18.5) | 122 (19.3) | 239 (18.9) |
| Some | 130 (20.6) | 140 (22.2) | 270 (21.4) |
| A fair amount - a great deal | 241 (38.1) | 205 (32.4) | 446 (35.3) |
| Missing | 144 (22.8) | 165 (26.1) | 309 (24.4) |
| **How much do you feel that you help other people in the gay male community when they need help?, n (%)** | | | |
| None - A little | 126 (19.9) | 138 (21.8) | 264 (20.9) |
| Some | 129 (20.4) | 138 (21.8) | 267 (21.1) |
| A fair amount - a great deal | 228 (36.1) | 191 (30.2) | 419 (33.1) |
| Missing | 149 (23.6) | 165 (26.1) | 314 (24.8) |
| **How much do you feel that you are a member of the gay male community?, n (%)** | | | |
| None - A little | 132 (20.9) | 147 (23.3) | 279 (22.1) |
| Some | 106 (16.8) | 98 (15.5) | 204 (16.1) |
| A fair amount - a great deal | 251 (39.7) | 217 (34.3) | 468 (37.0) |
| Missing | 143 (22.6) | 170 (26.9) | 313 (24.8) |
| **How many of your needs do you feel are met by the gay male community?, n (%)** | | | |
| None - a few | 285 (45.1) | 289 (45.7) | 574 (45.4) |
| About Half | 94 (14.9) | 86 (13.6) | 180 (14.2) |
| Most - All | 104 (16.5) | 89 (14.1) | 193 (15.3) |
| Missing | 149 (23.6) | 168 (26.6) | 317 (25.1) |
| **How much do you feel a part of the gay male community?, n (%)** | | | |
| None - A little | 137 (21.7) | 147 (23.3) | 284 (22.5) |
| Some | 105 (16.6) | 98 (15.5) | 203 (16.1) |
| A fair amount - a great deal | 243 (38.5) | 215 (34.0) | 458 (36.2) |
| Missing | 147 (23.3) | 172 (27.2) | 319 (25.2) |

**Supplemental Table 2. Adjusted^a^ Probit Estimates and 95% CIs for the Association of Multilevel Resilience Fac-rs, Depressive Symp-ms, and Loneliness at Time 1**

| **Fac-rs** | **Depression symp-ms at time 1** | **Loneliness at time 1** |
| --- | --- | --- |
|  | **Probit estimates (95% CI)** | |
| Global resilience | -0.39 (95% CI: -0.48 - -0.31) *** | -0.26 (95% CI: -0.34 - -0.18) *** |
| Relationship closeness | -0.37 (95% CI: -0.44 - -0.29) *** | -0.39 (95% CI: -0.46 - -0.32) *** |
| Relationship reliability | -0.37 (95% CI: -0.48 - -0.25) *** | -0.40 (95% CI: -0.50 - -0.30) *** |
| Relationship confidence | -0.38 (95% CI: -0.45 - -0.30) *** | -0.46 (95% CI: -0.53 - -0.39) *** |
| Relationship openness | -0.20 (95% CI: -0.29 - -0.12) *** | -0.28 (95% CI: -0.35 - -0.20) *** |
| Community belonging | -0.17 (95% CI: -0.24 - -0.10) *** | -0.27 (95% CI: -0.34 - -0.20) *** |
| Community helping | -0.24 (95% CI: -0.31 - -0.16) *** | -0.31 (95% CI: -0.38 - -0.24) *** |

^a^ Adjusted for by age, race and ethnicity, HIV status, enrollment wave, and education. *p<0.05; **p<0.01; ***p<0.0001
